# Supplementary material for: Comparing models of information transfer in the structural brain network and their relationship to functional connectivity: diffusion versus shortest path routing
Source: Brain Struct Funct. 2023 Feb 1;228(2):651–62. doi: 10.1007/s00429-023-02613-2 (PMC9944050; doi:10.1007/s00429-023-02613-2)
Supplement: Supplementary file 1 — Supplementary file1 (DOCX 41 kb) [file 429_2023_2613_MOESM1_ESM.docx]

**Supplementary Materials**

Supplementary Table 1. AAL individual-level linear regression analyses. Dependent variable was functional connectivity, and independent variables considered were mean first passage time (MFPT; log transformed), communicability (COM; log transformed), and shortest path length (SPL; log transformed).

| Variable | *R* | *R_adj_* | *p-*value |
| --- | --- | --- | --- |
| log(MFPT) | -.257 | -.257 | <.001 |
| log(COM) | .218 | .218 | <.001 |
| log(SPL) | -.251 | -.251 | <.001 |

Supplementary Table 2. AAL Individual-level multiple linear model 1, with dependent variable functional connectivity. *R^2^* = .072, *R_adj_^2^* = .072. Abbreviations include semi-partial correlation (SPC) and variance inflation factor (VIF).

| Effects | SPC | Estimate | Std. Error | *t*-value | *p*-value | VIF |
| --- | --- | --- | --- | --- | --- | --- |
| Intercept |  | .772 | 9.377*10^-4^ | 822.769 | <.001 |  |
| log(MFPT) | -.092 | -.056 | 2.931*10^-4^ | -191.660 | <.001 | 2.844 |
| log(SPL) | -.075 | -.047 | 3.059*10^-4^ | -155.079 | <.001 | 2.844 |

Supplementary Table 3. AAL Individual-level multiple linear model 2, with dependent variable functional connectivity. *R^2^* = .078, *R_adj_^2^* = .078. Abbreviations include semi-partial correlation (SPC) and variance inflation factor (VIF).

| Effects | SPC | Estimate | Std. Error | *t*-value | *p*-value | VIF |
| --- | --- | --- | --- | --- | --- | --- |
| Intercept |  | .771 | 9.347*10^-4^ | 825.186 | <.001 |  |
| log(MFPT) | -.103 | -.064 | 2.958*10^-4^ | -215.179 | <.001 | 2.915 |
| log(COM) | .077 | .011 | 6.906*10^-5^ | 161.222 | <.001 | 1.960 |
| log(SPL) | -.017 | -.013 | 3.724*10^-4^ | -34.832 | <.001 | 4.242 |

Supplementary Table 4. AAL individual-level partial least squares (PLS) regression. Dependent variable was functional connectivity, and independent variables considered were mean first passage time (MFPT; log transformed), communicability (COM; log transformed), and shortest path length (SPL; log transformed). Variance accounted for was *R^2^* = .077, *R_adj_^2^* = .077.

| Variable | Coefficient |
| --- | --- |
| log(MFPT) | -.039 |
| log(COM) | .019 |
| log(SPL) | -.017 |

Supplementary Table 5. AAL individual-level principal components analysis loadings for all 3 principal components. Variables considered were functional connectivity (FC), mean first passage time (MFPT; log transformed and multiplied by -1 so that more positive values indicate better connectivity), communicability (COM; log transformed), and shortest path length (SPL; log transformed and multiplied by -1 as with MFPT).

| Variable | PC1 | PC2 | PC3 |
| --- | --- | --- | --- |
| FC | -.278 | -.960 | .035 |
| -1*log(MFPT) | -.552 | .126 | -.609 |
| log(COM) | -.511 | .170 | .783 |
| -1*log(SPL) | -.597 | .185 | -.124 |

Supplementary Table 6. Brainnetome individual-level linear regression analyses. Dependent variable was functional connectivity, and independent variables considered were mean first passage time (MFPT; log transformed), communicability (COM; log transformed), and shortest path length (SPL; log transformed).

| Variable | *R* | *R_adj_* | *p-*value |
| --- | --- | --- | --- |
| log(MFPT) | -.140 | -.140 | <.001 |
| log(COM) | .168 | .168 | <.001 |
| log(SPL) | -.166 | -.166 | <.001 |

Supplementary Table 7. Brainnetome individual-level multiple linear model 1, with dependent variable functional connectivity. *R^2^* = .028, *R_adj_^2^* = .028. Abbreviations include semi-partial correlation (SPC) and variance inflation factor (VIF).

| Effects | SPC | Estimate | Std. Error | *t*-value | *p*-value | VIF |
| --- | --- | --- | --- | --- | --- | --- |
| Intercept |  | .517 | 3.306*10^-4^ | 1564.054 | <.001 |  |
| log(MFPT) | -.016 | -.008 | 8.567*10^-5^ | -91.187 | <.001 | 2.579 |
| log(SPL) | -.091 | -.054 | 1.068*10^-4^ | -503.269 | <.001 | 2.579 |

Supplementary Table 8. Brainnetome individual-level multiple linear model 2, with dependent variable functional connectivity. *R^2^* = .034, *R_adj_^2^* = .034. Abbreviations include semi-partial correlation (SPC) and variance inflation factor (VIF).

| Effects | SPC | Estimate | Std. Error | *t*-value | *p*-value | VIF |
| --- | --- | --- | --- | --- | --- | --- |
| Intercept |  | .500 | 3.317*10^-4^ | 1505.702 | <.001 |  |
| log(MFPT) | -.032 | -.016 | 8.717*10^-5^ | -180.304 | <.001 | 2.689 |
| log(COM) | .081 | .008 | 1.814*10^-5^ | 449.561 | <.001 | 1.896 |
| log(SPL) | -.025 | -.018 | 1.322*10^-4^ | -139.992 | <.001 | 3.978 |

Supplementary Table 9. Brainnetome individual-level partial least squares (PLS) regression. Dependent variable was functional connectivity, and independent variables considered were mean first passage time (MFPT; log transformed), communicability (COM; log transformed), and shortest path length (SPL; log transformed). Variance accounted for was *R^2^* = .034, *R_adj_^2^* = .034.

| Variable | Coefficient |
| --- | --- |
| log(MFPT) | -.009 |
| log(COM) | .023 |
| log(SPL) | -.014 |

Supplementary Table 10. Brainnetome individual-level principal components analysis loadings for all 3 principal components. Variables considered were functional connectivity (FC), mean first passage time (MFPT; log transformed and multiplied by -1 so that more positive values indicate better connectivity), communicability (COM; log transformed), and shortest path length (SPL; log transformed and multiplied by -1 as with MFPT).

| Variable | PC1 | PC2 | PC3 |
| --- | --- | --- | --- |
| FC | -.202 | -.977 | -.074 |
| -1*log(MFPT) | -.556 | .158 | -.609 |
| log(COM) | -.520 | .046 | .785 |
| -1*log(SPL) | -.617 | .139 | -.088 |

Supplementary Table 11. AAL split-half linear regression analyses. Model was trained on a training set and then tested on a testing set. Dependent variable was functional connectivity, and independent variables considered were mean first passage time (MFPT; log transformed), communicability (COM; log transformed), and shortest path length (SPL; log transformed).

|  |  |  | Train | | Test | | |  |
| --- | --- | --- | --- | --- | --- | --- | --- | --- |
| Variable |  | *R* | *R_adj_* | *p-*value | *R* | *R_adj_* | *p-*value | |
| log(MFPT) |  | -.374 | -.373 | <.001 | -.378 | -.378 | <.001 | |
| log(COM) |  | .315 | .314 | <.001 | .317 | .316 | <.001 | |
| log(SPL) |  | -.377 | .376 | <.001 | -.382 | -.381 | <.001 | |

Supplementary Table 12. AAL split-half multiple linear model 1. Model was trained on a training set and then tested on a testing set, with dependent variable functional connectivity. Variance accounted for in training set was *R^2^* = .155, *R_adj_^2^* = .155, and in testing set was *R^2^* = .159, *R_adj_^2^* = .159. Abbreviations include semi-partial correlation (SPC) and variance inflation factor (VIF).

| Effects | SPC | Estimate | Std. Error | *t*-value | *p*-value | VIF |
| --- | --- | --- | --- | --- | --- | --- |
| Intercept |  | .838 | .023 | 36.235 | <.001 |  |
| log(MFPT) | -.115 | -.059 | .007 | -7.947 | <.001 | 2.946 |
| log(SPL) | -.126 | -.066 | .008 | -8.657 | <.001 | 2.946 |

Supplementary Table 13. AAL split-half multiple linear model 2. Model was trained on a training set and then tested on a testing set, with dependent variable functional connectivity. Variance accounted for in training set was *R^2^* = .164, *R_adj_^2^* = .163, and in testing set was *R^2^* = .167, *R_adj_^2^* = .166. Abbreviations include semi-partial correlation (SPC) and variance inflation factor (VIF).

| Effects | SPC | Estimate | Std. Error | *t*-value | *p*-value | VIF |
| --- | --- | --- | --- | --- | --- | --- |
| Intercept |  | .844 | .023 | 36.644 | <.001 |  |
| log(MFPT) | -.138 | -.074 | .008 | -9.529 | <.001 | 3.256 |
| log(COM) | .091 | .012 | .002 | 6.268 | <.001 | 2.494 |
| log(SPL) | -.029 | -.021 | .011 | -1.988 | .047 | 5.605 |

Supplementary Table 14. AAL split-half partial least squares (PLS) regression. Model was trained on a training set and then tested on a testing set. Dependent variable was functional connectivity, and independent variables considered were mean first passage time (MFPT; log transformed), communicability (COM; log transformed), and shortest path length (SPL; log transformed). Variance accounted for in training set was *R^2^* = .163, *R_adj_^2^* = .162, and in testing set was *R^2^* = .166, *R_adj_^2^* = .166.

| Variable | Coefficient |
| --- | --- |
| log(MFPT) | -.037 |
| log(COM) | .018 |
| log(SPL) | -.024 |

Supplementary Table 15. Brainnetome split-half linear regression analyses. Model was trained on a training set and then tested on a testing set. Dependent variable was functional connectivity, and independent variables considered were mean first passage time (MFPT; log transformed), communicability (COM; log transformed), and shortest path length (SPL; log transformed).

|  |  | Train | | | Test | | |
| --- | --- | --- | --- | --- | --- | --- | --- |
| Variable | *R* | *R_adj_* | *p-*value | *R* | | *R_adj_* | *p-*value |
| log(MFPT) | -.230 | -.230 | <.001 | -.233 | | -.233 | <.001 |
| log(COM) | .268 | .268 | <.001 | .272 | | .272 | <.001 |
| log(SPL) | -.248 | -.248 | <.001 | -.252 | | -.251 | <.001 |

Supplementary Table 16. Brainnetome split-half multiple linear model 1. Model was trained on a training set and then tested on a testing set, with dependent variable functional connectivity. Variance accounted for in training set was *R^2^* = .066, *R_adj_^2^* = .066, and in testing set was *R^2^* = .068, *R_adj_^2^* = .068. Abbreviations include semi-partial correlation (SPC) and variance inflation factor (VIF).

| Effects | SPC | Estimate | Std. Error | *t*-value | *p*-value | VIF |
| --- | --- | --- | --- | --- | --- | --- |
| Intercept |  | .606 | .008 | 73.398 | <.001 |  |
| log(MFPT) | -.066 | -.024 | .002 | -11.805 | <.001 | 2.295 |
| log(SPL) | -.115 | -.051 | .002 | -20.588 | <.001 | 2.295 |

Supplementary Table 17. Brainnetome split-half multiple linear model 2. Model was trained on a training set and then tested on a testing set, with dependent variable functional connectivity. Variance accounted for in training set was *R^2^* = .089, *R_adj_^2^* = .089, and in testing set was *R^2^* = .091, *R_adj_^2^* = .091. Abbreviations include semi-partial correlation (SPC) and variance inflation factor (VIF).

| Effects | SPC | Estimate | Std. Error | *t*-value | *p*-value | VIF |
| --- | --- | --- | --- | --- | --- | --- |
| Intercept |  | .552 | .008 | 65.820 | <.001 |  |
| log(MFPT) | -.112 | -.044 | .002 | -20.299 | <.001 | 2.573 |
| log(COM) | .151 | .015 | .001 | 27.430 | <.001 | 2.718 |
| log(SPL) | .034 | .022 | .004 | 6.204 | <.001 | 5.049 |

Supplementary Table 18. Brainnetome split-half partial least squares (PLS) regression. Model was trained on a training set and then tested on a testing set. Dependent variable was functional connectivity, and independent variables considered were mean first passage time (MFPT; log transformed), communicability (COM; log transformed), and shortest path length (SPL; log transformed). Variance accounted for in training set was *R^2^* = .086, *R_adj_^2^* = .086 and in testing set was *R^2^* = .088, *R_adj_^2^* = .088.

| Variable | Coefficient |
| --- | --- |
| log(MFPT) | -.015 |
| log(COM) | .034 |
| log(SPL) | -.003 |

Supplementary Table 19. AAL null model mean distance from empirical PC vectors in PCA analysis, using a 3-dimensional distance calculation with the 3 PCs as dimensions. Bootstrapped 95% confidence intervals (95% CI) indicate that all measures were significantly different than the empirical PCs.

| Variable | Mean Distance | 95% CI | |
| --- | --- | --- | --- |
| FC | .673 | .634 | .717 |
| -1*log(MFPT) | .915 | .881 | .946 |
| log(COM) | .838 | .804 | .870 |
| -1*log(SPL) | .980 | .948 | 1.010 |

Supplementary Table 20. Brainnetome null model mean distance from empirical PC vectors in PCA analysis, using a 3-dimensional distance calculation with the 3 PCs as dimensions. Bootstrapped 95% confidence intervals (95% CI) indicate that all measures were significantly different than the empirical PCs.

| Variable | Mean Distance | 95% CI | |
| --- | --- | --- | --- |
| FC | .490 | .489 | .491 |
| -1*log(MFPT) | 1.190 | 1.188 | 1.191 |
| log(COM) | 1.065 | 1.064 | 1.066 |
| -1*log(SPL) | 1.269 | 1.269 | 1.270 |
